# Performance comparison of three DNA extraction kits on human whole-exome data from formalin-fixed paraffin-embedded normal and tumor samples

**DOI:** 10.1371/journal.pone.0195471

**Published:** 2018-04-05

**Authors:** Eric Bonnet, Marie-Laure Moutet, Céline Baulard, Delphine Bacq-Daian, Florian Sandron, Lilia Mesrob, Bertrand Fin, Marc Delépine, Marie-Ange Palomares, Claire Jubin, Hélène Blanché, Vincent Meyer, Anne Boland, Robert Olaso, Jean-François Deleuze

**Affiliations:** 1 Centre National de Recherche en Génomique Humaine, Institut de Biologie François Jacob, Direction de la Recherche Fondamentale, CEA, Evry, France; 2 LabEx GenMed, Evry, France; 3 Centre d’Etude du Polymorphisme Humain, Fondation Jean Dausset, Paris, France; 4 Centre de REFérence, d’Innovation, d’eXpertise et de transfert (CREFIX), Evry, France; Pennsylvania State University, UNITED STATES

## Abstract

Next-generation sequencing (NGS) studies are becoming routinely used for the detection of novel and clinically actionable DNA variants at a pangenomic scale. Such analyses are now used in the clinical practice to enable precision medicine. Formalin-fixed paraffin-embedded (FFPE) tissues are still one of the most abundant source of cancer clinical specimen, unfortunately this method of preparation is known to degrade DNA and therefore compromise subsequent analysis. Some studies have reported that variant detection can be performed on FFPE samples sequenced with NGS techniques, but few or none have done an in-depth coverage analysis and compared the influence of different state-of-the-art FFPE DNA extraction kits on the quality of the variant calling. Here, we generated 42 human whole-exome sequencing data sets from fresh-frozen (FF) and FFPE samples. These samples include normal and tumor tissues from two different organs (liver and colon), that we extracted with three different FFPE extraction kits (QIAamp DNA FFPE Tissue kit and GeneRead DNA FFPE kit from Qiagen, Maxwell^™^ RSC DNA FFPE Kit from Promega). We determined the rate of concordance of called variants between matched FF and FFPE samples on all common variants (representing at least 86% of the total number of variants for SNVs). The concordance rate is very high between all matched FF / FFPE pairs, with equivalent values for the three kits we analyzed. On the other hand, when looking at the difference between the total number of variants in FF and FFPE, we find a significant variation for the three different FFPE DNA extraction kits. Coverage analysis shows that FFPE samples have less good indicators than FF samples, yet the coverage quality remains above accepted thresholds. We detect limited but statistically significant variations in coverage indicator values between the three FFPE extraction kits. Globally, the GeneRead and QIAamp kits have better variant calling and coverage indicators than the Maxwell kit on the samples used in this study, although this kit performs better on some indicators and has advantages in terms of practical usage. Taken together, our results confirm the potential of FFPE samples analysis for clinical genomic studies, but also indicate that the choice of a FFPE DNA extraction kit should be done with careful testing and analysis beforehand in order to maximize the accuracy of the results.

## Introduction

Next-generation sequencing (NGS) approaches have proven to be a cost-effective and relevant method for the identification of novel and clinically actionable variants across many genes in a single test [1–7]. NGS is nowadays commonly used in clinical molecular diagnostic for the detection of germline and somatic variants [4–6, 8–10]. NGS can be used to detect the full range of DNA variations, i.e. single-nucleotide variants (SNVs), insertions/deletions (INDELs), translocations and copy-number changes. The main advantage of NGS over traditional techniques, such as Sanger sequencing, is the greater level of multiplexing of genes that NGS can offer, along with the ability to detect mutations across an entire gene as opposed to PCR based methods that focus on specific single nucleotide variants.

Using NGS approaches for routine clinical testing implicates testing for the various type of samples that may be used in the laboratory and that should be generated with minimally invasive techniques for the patient. Most molecular tests are performed on fresh (and/or frozen) tissues (e.g. blood samples or biopsies), mainly because this minimizes the risk of DNA degradation in the sample. However, in most clinical molecular pathology settings, FF tissues are rare, due to the complexities of the logistic chain for the preparation, collection and storage of such samples. Instead, FFPE is the method of choice (and sometimes the gold standard method) for clinicians. FFPE specimen are much easier to prepare and to store, but it is well established that formalin fixation results in DNA damage. Formaldehyde reacts with DNA and proteins, resulting in DNA-DNA, DNA-RNA, and DNA-protein molecules that are covalently linked by methylene bridges. Formaldehyde is also known to induce oxidation and deamination reactions and the formation of cyclic bases derivatives. These chemical modifications have the potential to alter molecular testing through inhibition of enzymatic reparation of DNA or direct changes at single base or sequence levels. Furthermore, crosslinks lead to DNA fragmentation that render sequencing and analysis even more complicated [11–16].

The dramatic decrease in NGS related costs associated with the demonstrated capability of detection of clinically actionable targets and affordable large-scale computational power have triggered the creation of large projects (such as the precision medicine initiative [17]) aiming at bringing the benefits of large scale genomic analyses to patients suffering from a variety of diseases. In this context, there is a strong interest in analyses based on FFPE samples, due to their large abundance in clinical biobanks.

A number of studies have already established that NGS can be performed with DNA from FFPE samples. For instance, in one of the earliest attempts, Schweiger and colleagues showed that short reads sequencing could be applied to FFPE samples to detect genomic variations and copy number alterations in normal and tumor breast tissue, in spite of using low coverage [18]. A more recent study examined hybridization-capture of twenty-seven cancer-related genes with NGS on paired FF and FFPE samples, detecting a high degree of concordance and agreement between them [19]. Several studies have assessed the performance of various forms of DNA sequencing (whole-exome, whole-genome, targeted exon sequencing), and even tried RNA sequencing on FFPE samples [20–23]. Those studies have found a high degree of concordance between FF and FFPE samples and concluded that NGS-based analysis of FFPE samples could be used in both prospective and retrospective studies with the possibility to uncover clinically important genes. It is also worth noticing that some groups specifically tackled the question of the effect of FFPE storage time on the quality of the results. For instance, Hegegaard and colleagues found a high concordance of variants in FF and FFPE samples stored for fewer than three years [20]. Carrick et al. [23] analyzed a set of samples stored from 3 to 32 years and reported that 90% of the samples could provide data with sufficient quantity and quality for data mining regardless of storage time, although specimens stored for longer periods of time had significantly lower coverage of the target regions and lower average read depth. At last there is at least one study that used FFPE material for causal variants discovery: Bagnall and colleagues found significant variants in two genes associated to sudden death syndrome by performing exome sequencing on FFPE samples [24].

However, few studies analyzed in details the sequencing coverage for matched FF/FFPE samples and studied the impact of recent state-of-the-art DNA extraction kits that are compatible with systems allowing an automation of sample preparation. For instance, Janecka and colleagues [25] compared eight different commercial kits for preparing DNA from FFPE samples, but only analyzed the quality of the DNA obtained and did not perform variant analysis. More recently, Bonfiglio et al. [26] compared two solutions-based exome capture technologies by comparing coverage and variant detection, but did not address the problem of different extraction kits. Astolfi et al. [27] performed whole-exome sequencing on four gastrointestinal stromal tumor samples either extracted from FF or FFPE, and analyzed the quality of the DNA extracted and the variants called on the samples. They concluded on the feasibility of WES based analysis of FFPE samples compared to FF, but they did not test the influence of FFPE DNA extraction kits on the results. Heydt and colleagues [28] evaluated five automated DNA extraction systems and five DNA quantification systems on FFPE samples, focusing mainly the analysis on DNA quality related parameters, but they did not include in-depth coverage analysis and whole-exome sequencing variant calling analysis.

High-quality DNA extraction with automated sample preparation is of course an important point to consider, that may facilitate the implementation of routine NGS based analysis of FFPE samples for large scale, high-throughput clinical precision medicine projects in the near future. In this study, we performed whole-exome deep sequencing of 42 human FF and FFPE samples, including tumor and normal samples extracted from liver and colon tissues. DNA was extracted from FFPE samples using three different commercial kits, namely the QIAamp DNA FFPE Tissue kit (QIAGEN), the GeneRead DNA FFPE kit (QIAGEN) and the Maxwell RSC DNA FFPE kit (Promega). We selected those kits as robust and well-established methods for DNA extraction from FFPE samples and also for their ability to be included in automated systems for sample preparation. Extraction of FF samples was performed with either a QIAamp DNA Micro Kit or Maxwell^™^ RSC Blood DNA kit. After sequencing, we did a detailed sequencing coverage analysis for all samples, and then finally performed variants analysis. At each step, we compared FF and FFPE conditions for matched pairs of samples, and checked the effects of the three different extraction kits on FFPE samples. FFPE artifacts and tumor-specific variants annotation were also analyzed.

## Materials and methods

### FF and FFPE samples

We ordered matched FFPE and FF samples from company AMS BIOTECHNOLOGY EUROPE Ltd. We included tissues from two different organs (liver and colon) in this study. For each tissue, all samples come from the same individual and include both normal and tumor samples from fresh-frozen and formalin-fixed, paraffin-embedded tissues. All the samples were frozen or formalin-fixed in 2013 and 2015. As we processed the samples in 2016, we therefore have a storage time of maximum three years for FF and FFPE. In total we analyzed 42 samples, of which 26 FFPE (10 processed with the Qiagen GeneRead DNA FFPE kit, 8 processed with the Promega Maxwell RSC DNA FFPE kit and 8 processed with the Qiagen QIAamp DNA FFPE Tissue kit) and 16 FF (8 processed with the Maxwell RSC Blood DNA kit, 8 processed with the QIAamp DNA Micro kit). For the analysis, we build a list of sample pairs, associating FF and FFPE samples according to the tissue type and extraction kits. As we have less FF samples than FFPE, the FF samples were repeatedly paired to the FFPE samples extracted with different kits. Finally we have a list of 26 sample pairs, of which 10 FF/QIAamp samples paired with 10 FFPE/GeneRead samples, 8 FF/Maxwell samples paired with 8 FFPE/Maxwell samples and 8 FF/QIAamp samples paired with 8 FFPE/QIAamp samples (see S1 and S2 Tables for the details).

### Purification and quality control of DNA from FF and FFPE samples

All the DNA extractions were made from 10 *μ*m thick sample slices. For FFPE samples, we used the QIAamp DNA FFPE Tissue kit (QIAGEN), the GeneRead DNA FFPE kit (QIAGEN) and the Maxwell RSC DNA FFPE kit (Promega). For the FF samples we used the QIAamp DNA Micro Kit (QIAGEN) or the Maxwell^™^ RSC Blood DNA Kit (Promega). All samples were extracted after an overnight proteinaseK digestion step at 65°C. All Maxwell^™^ extractions were performed according to the manufacturer’s protocols, on a Maxwell RSC device from Promega. QIAGEN extractions were also performed according to the manufacturer’s instructions, including systematically a RNAse treatment and an optimized elution step. After extraction, all DNA samples were quantified in fluorescence and in duplicate, using Quant-iT^™^ dsDNA Assay Kits (Invitrogen). The quality of the DNA for all samples has been assessed by loading an aliquot of ∼ 20 ng on a TapeStation 4200 from Agilent to determine the DNA Integrity Number (DIN).

### Sequencing of DNA exome libraries and analysis

Exomes were captured using the Agilent Sureselect All Exons Human V5 kit (Agilent Technologies, santa Clara, CA, USA) according to manufacturer’s instructions, with an input of 200 ng. Final libraries were sequenced on a HiSeq2000 with 100 bp paired-end reads (samples were pooled by three on each lane).

### Bioinformatics analysis

The reads were mapped to the human genome (GRCh37) using BWA 0.7.12 [29]. Picard Tools 2.6.0 was used to flag duplicate reads and we applied the GATK for indel realignment, base quality score recalibration, SNPs and indels calling using the Haplotype Caller algorithm across all samples simultaneously according to the GATK Best Practices recommendations [30]. After calling we filtered the vcf files for variants having a coverage ≥ 13 and a mapping quality ≥ 43 (as done by Munchel and colleagues in their study [22]). For the coverage analysis, we used SAMtools 1.3.1 [31], BEDtools 2.21.0 [32] and a custom Python script to generate all the statistics. For the analysis of variants, we used Picard Tools to select variants on their quality and discriminate between SNPs and indels. A custom script was also used to compare the variants between FF and FFPE samples. For the somatic analysis, we followed a protocol described in [22]. Briefly, we selected SNVs for colon and liver tumors that were not present in normal samples. This selection was done for FF and FFPE conditions separately, then we counted how many of those tumor-specific variants were in common between the FF and FFPE conditions. All the variants were further annotated with snpEff [33], the COSMIC catalog of somatic mutations in cancer [34] and the KEGG database of biological pathways [35]. The VAF (Variant Allele Frequency) values were calculated as the ratio between the depth of the alternative allele (AD) and the total depth (DP). The AD and DP values were extracted from the VCF files. For the average gene coverage, we used the tool sambamba [36] to calculate the read coverage for all the position of a given gene from the alignments files (option “depth base”).

## Results

### DNA quality analysis

**Fig 1:**
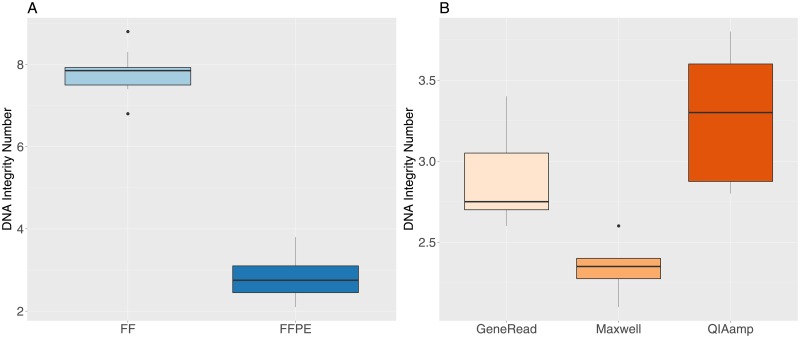
DNA Integrity Number (DIN) values for FF and FFPE samples. A: FF and FFPE samples. B: FFPE samples grouped by extraction method.

We checked the DNA quality for all samples by measuring the DNA Integrity Number (DIN, the equivalent of the RNA Integrity Number [37] for DNA). Unsurprisingly, the DIN values are much lower in FFPE compared to FF samples (Fig 1A), and the difference is highly significant (t-test t = 34.9, df = 33.9, p-value = 2.2e-16). Lower FFPE DIN values indicate more fragmented DNA and a lower molecular weight for those samples. Furthermore, we find a significant difference for the DIN values between the three extraction methods for the FFPE samples (Fig 1B, one-way anova, F-value = 19.7, df = 2, p-value = 1.03e-5). Samples treated with the Maxwell kit have the lowest values, followed by GeneRead samples and finally the QIAamp samples.

**Fig 2:**
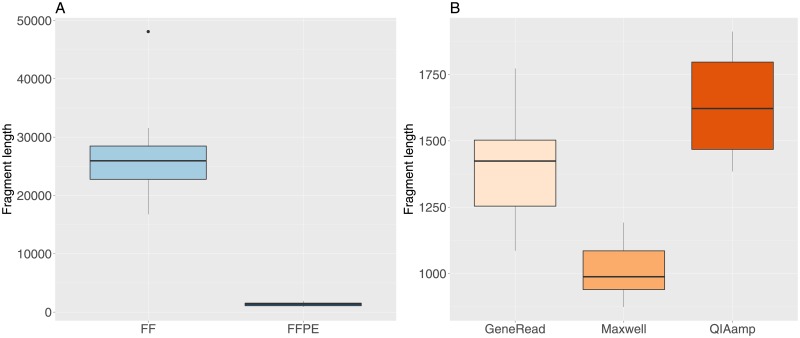
DNA fragment length values for FF and FFPE samples. A: FF and FFPE samples. B: FFPE samples grouped by extraction method.

The DNA fragment length was obtained from the readouts of the DIN analysis (length in base pairs of the main peak). The median fragment length is very significantly shorter in FFPE (1368 bp) compared to FF samples (25946 bp, Fig 2A, t-test t = 14.3, df = 15.036, p-value = 3.7e-10). There are also significant differences between the median fragment length for the three different extraction methods (Fig 1B, with increasing fragment length for the Maxwell (median value 988 bp), GeneRead (1424 bp) and QIAamp (1622 bp) kits (one-way anova, F-value = 24.15, df = 2, p-value = 2.2e-6).

### Coverage analysis

**Fig 3:**
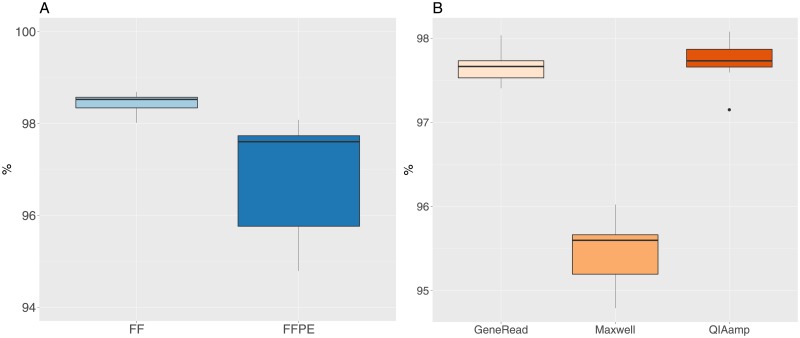
Percentage of positions having a coverage greater then or equal to 30X. A: FF and FFPE samples. B: FFPE samples grouped by extraction method.

Initial analysis of the mapped reads revealed an unequal number of reads between the samples (median 123M, IQR 27M). In order to be able to compare the samples, we re-sampled all the files to 80M reads. One sample was eliminated from the study, having a value of 73M reads, which is below our usual quality threshold. Re-sampled files were mapped to the human genome, with a median percentage of reads mapped of 99.90% (minimum value 99.73, maximum value 99.96). The median percentage of reads mapping outside the target regions (as defined by the exome capture kit) is 20.3% (min. 18.1%, max. 23.2%), which is within the values that are usually accepted for human whole-exome analysis. The percentage of positions having a coverage greater than or equal to 30X is very high for both FF and FFPE samples (Fig 3A, mean values of 98.4 and 97% respectively), but the difference between the two groups is significant (t-test t = 6.4, df = 28.49, p-value = 4.8e-7). For FFPE samples, there is a significant difference between the three extraction methods (one-way anova F = 165, df = 2, p-value = 2.3e-14), with the Maxwell kit having less coverage than the other two methods (Fig 3B).

**Fig 4:**
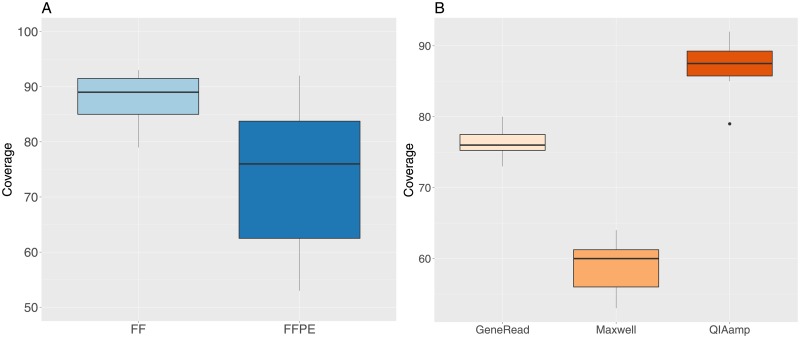
Median coverage values for FF and FFPE samples. A: FF and FFPE samples. B: FFPE samples grouped by extraction method.

The mean median coverage value for all samples is 80X (Fig 4A), but there is a significant difference (t-test t = 5.03, df = 36.8, p-value = 1.3e-05) between FF (mean value of 87.6X) and FFPE (mean value of 74.3X) samples. The median coverage is also significantly different between the the three extraction methods (one-way anova F = 149.7, df = 2, p-value = 6.5e-14), with increasing values for the Maxwell, GeneRead and QIAamp methods (Fig 4B).

**Fig 5:**
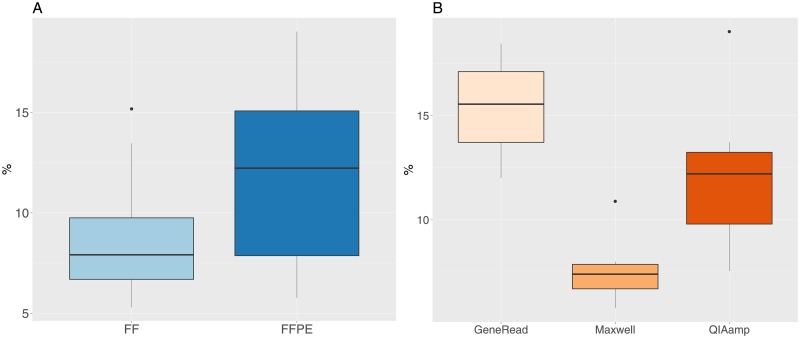
Percentage of duplicated reads for FF and FFPE samples. A: FF and FFPE samples. B: FFPE samples grouped by extraction method.

The percentage of duplicated reads is an important indicator of the sequencing quality for further analysis. We have a median value of 10% for all samples (Fig 5A), but the values are significantly different for FF (median value 7.9%) and FFPE (median value 12.2%, t-test t = -3.06, df = 37.7, p-value = 0.004). For FFPE values only, there is a significant difference between the three extraction methods (Fig 5B, one-way anova F = 21, df = 2, p-value = 6.4e-6). The lowest level is observed for the Maxwell method, then follows the QIAamp method and finally the GeneRead method who has the highest level of duplicated reads.

**Fig 6:**
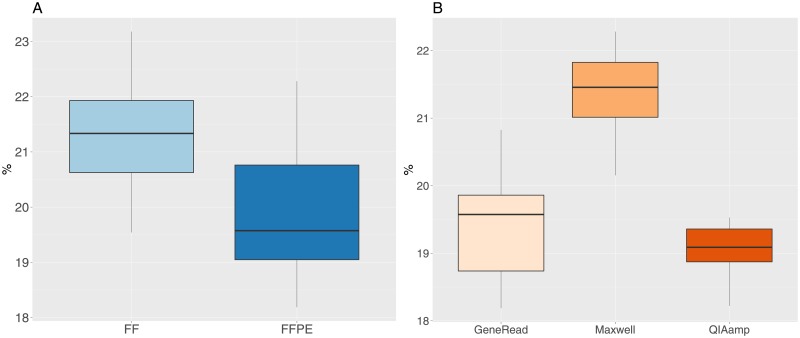
Percentage of reads mapping outside target regions. A: FF and FFPE samples. B: FFPE samples grouped by extraction method.

The percentage of reads mapping outside the target regions is slightly lower in FFPE (median value 19.6) compared to FF samples (median value 21.3, t-test t = 4.1, df = 35.1, p-value = 0.0002, Fig 6A). For the FFPE values, we observe a significant difference between the three extraction methods (Fig 6B, one-way anova F = 27, df = 2, p-value = 9.2e-7). The Maxwell kit has the highest median value (21.4) for the percentage of reads mapping outside the target regions, while the QIAamp and GeneRead have lower values (19.1 and 19.6 respectively). However, the percentage values for the three kits are quite close.

### Variant analysis and FFPE artifacts

To analyze the potential sequencing artifacts and biases induced by the FFPE treatment, we used 25 matched pairs of both normal and tumor samples of liver and colon tissue. After whole exome sequencing, we characterized all pairs by calling both single nucleotide (SNVs) and small insertion-deletion (INDELs) events, and filtering out low quality calls (see Methods). In order to assess the reliability of base calls from FFPE samples, we determined the common positions between the FF and FFPE lists of variants, and then we used calls from the FF samples as the reference and classified FFPE base calls as concordant if they are the same or discordant if they are not the same. For each pair we can calculate a concordance rate as the number of concordant bases divided by the number of common positions.

The results for SNVs and INDELs are shown in Tables 1 and 2. The average number of variants is significantly lower in FFPE for both SNVs and INDELs (mean values of 40938 SNV variants for FF, 39748 for FPPE, t-test t = 4.2, df = 25.1, p-value = 0.0003, mean values of 3680 INDELs for FF, 3370 for FFPE, t-test t = 5.2, df = 26.7, p-value = 1.7e-05). The number of common positions for filtered base calls represent minimum 86% of the total number of positions for SNVs, and a minimum of 67% for INDELs. The percentage of concordance for SNVs on common positions is minimum 99.98%, and 98.44% for INDELs.

**Table 1:** Single nucleotide variants (SNVs) analysis between FF and FFPE sample pairs. NFF: Number of SNV in FF samples, NFFPE: number of SNV in FFPE samples, NPos: number of common positions between FF and FFPE samples, Nco: number of concordant positions, Ndi: number of discordant positions, P: concordance rate (Nco/NPos * 100).

Pair	NFF	NFFPE	NPos	Nco	Ndi	P
1	41008	40165	39778	39772	6	99.98
2	40902	40157	39691	39685	6	99.98
3	40950	40361	39913	39906	7	99.98
4	41078	40011	39643	39639	4	99.99
5	40705	40295	39355	39351	4	99.99
6	40999	40134	39570	39567	3	99.99
7	40571	40481	39634	39629	5	99.99
8	40568	40623	39568	39564	4	99.99
9	41008	40716	40075	40068	7	99.98
10	40902	40764	40048	40044	4	99.99
11	40902	40712	40004	40001	3	99.99
12	40950	40712	40094	40089	5	99.99
13	41078	40788	40102	40098	4	99.99
14	41078	40779	40116	40110	6	99.99
15	40705	40794	39631	39626	5	99.99
16	40999	40852	40046	40042	4	99.99
17	40571	40586	39473	39468	5	99.99
18	40568	41009	39848	39844	4	99.99
19	41239	37396	36851	36846	5	99.99
20	41201	37834	37239	37236	3	99.99
21	41115	37721	37185	37179	6	99.98
22	41189	36215	35751	35745	6	99.98
23	40963	38097	37588	37582	6	99.98
24	41074	38132	37577	37572	5	99.99
25	41133	38369	37860	37858	2	99.99

**Table 2:** Insertion-deletion events (INDELs) analysis between FF and FFPE sample pairs. NFF: Number of INDELs in FF samples, NFFPE: number of INDELs in FFPE samples, NPos: number of common positions between FF and FFPE samples, Nco: number of concordant positions, Ndi: number of discordant positions, P: percentage of concordance Nco/NPos.

Pair	NFF	NFFPE	NPos	Nco	Ndi	P
1	3609	3402	3176	3140	36	98.87
2	3612	3369	3169	3122	47	98.52
3	3610	3435	3193	3154	39	98.78
4	3624	3428	3168	3122	46	98.55
5	3762	3445	3178	3136	42	98.68
6	3770	3446	3212	3170	42	98.69
7	3749	3587	3315	3276	39	98.82
8	3712	3589	3239	3203	36	98.89
9	3609	3568	3258	3210	48	98.53
10	3612	3589	3267	3221	46	98.59
11	3612	3538	3240	3196	44	98.64
12	3610	3588	3267	3216	51	98.44
13	3624	3565	3251	3206	45	98.62
14	3624	3567	3271	3235	36	98.90
15	3762	3611	3293	3245	48	98.54
16	3770	3614	3336	3292	44	98.68
17	3749	3629	3311	3268	43	98.70
18	3712	3711	3346	3310	36	98.92
19	3723	2931	2740	2703	37	98.65
20	3770	2999	2826	2797	29	98.97
21	3763	2957	2791	2759	32	98.85
22	3732	2736	2593	2570	23	99.11
23	3600	2952	2779	2756	23	99.17
24	3635	2972	2788	2752	36	98.71
25	3651	3036	2839	2810	29	98.98

**Fig 7:**
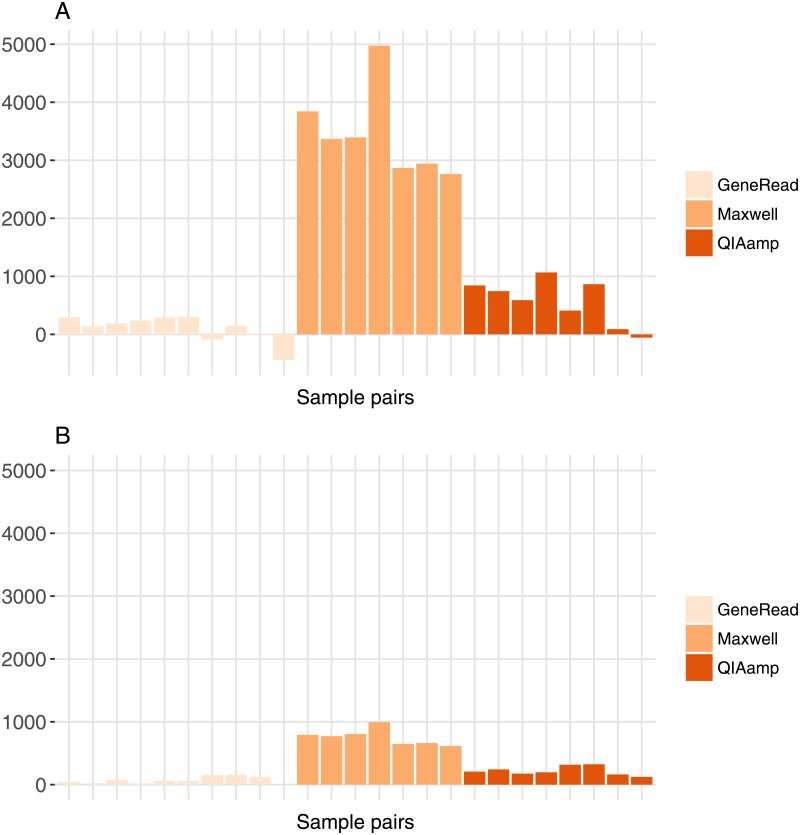
Difference in number of variants between FF and FFPE samples for all matched FF/FFPE pairs. A: SNVs. B: INDELs.

To evaluate the impact of the different extraction kits, we calculated the difference in number of variants for each pair of matched FF/FFPE samples for SNVs and INDELs (Fig 7A and 7B). There is a significant variation in difference values for the three extraction kits for both SNVs (one-way anova, F-Value = 108.6, df = 2, p-value = 3.9e-12) and INDELs (one-way anova, F-Value = 135.5, df = 2, p-value = 4.3e-13). We can see on the graph that difference values are low for GeneRead and QIAamp, while values for Maxwell are much higher, especially for SNVs. The GeneRead kit has the lowest median value, which might be due to the fact that this kit is designed to minimize the number of artifacts induced in FFPE samples (by enzymatic correction). While the difference values for SNVs represent on average 1% of the total number of variants for GeneRead and QIAamp, they increase to around 10% of the total number of variants for the Maxwell kit (Fig 7A). The profile of the variations is similar for INDELs (Fig 7B).

**Fig 8:**
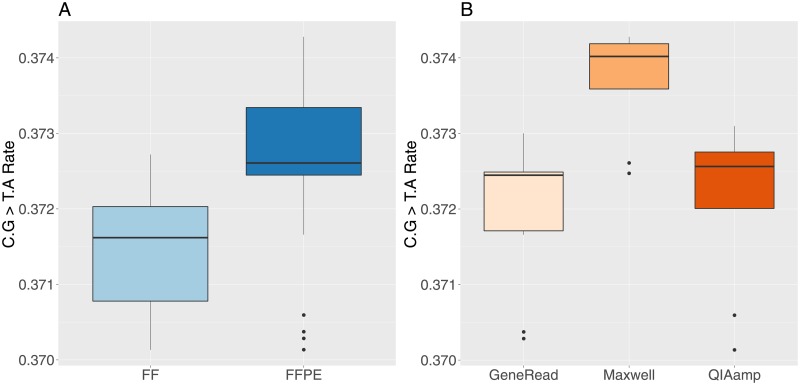
Rate of SNVs C.G > T.A substitution in FF and FFPE samples. A: FF and FFPE samples. B: FFPE samples grouped by extraction method.

FFPE DNA has been shown to have artifacts created by formalin fixation and sample preparation that trigger enhanced cytosine deamination [38–40]. These artifacts show up as non-reproducible C > T or G > A (C.G > T.A) substitutions. We therefore analyzed combined C > T, G > A substitutions in SNV variants for all samples. As expected, the number of substitutions is higher in FFPE samples (Fig 8A, t-test t = -3.8, df = 37.9, p-value = 0.0005) and the values are slightly higher in Maxwell treated samples, followed by QIAamp and GeneRead (Fig 8B, one-way anova, F-value = 9.5, df = 2, p-value = 0.009). The GeneRead kit has the lowest values for the C > T, G > A substitution rates, which is most likely due to the artifact correction capabilities included in this kit (by enzymatic activity). Although the difference is statistically significant, it is worth noticing that the increase in rate value between FF and FFPE, and between the extraction methods remains small in absolute value. As shown on Fig 8, the median value difference between FFPE and FF C.G > T.A rates is equal to 0.0098 (≈ 1%, the median value differences between Maxwell and GeneRead for FFPE data only is equal to 0.0015, the median value differences between Maxwell and GeneRead for FFPE data only is equal to 0.00011, i.e. ≈ 0.01%).

Since we had matched normal and tumor tissues, we performed somatic mutation analysis to evaluate the potential impact and interest of the SNVs called. Upon subtraction of variants present in matched normal samples, we found 394 putative somatic variants in liver FF samples, 333 in liver FFPE samples, 436 in colon FF samples and 458 in colon FFPE samples (S3 Table). We observe that a total of 165 tumor-specifc SNVs for colon show overlap between FF and FFPE (representing 38% and 36% of tumor-specific FF and FFPE SNVs respectively, see S3 Table) and that 53 tumor-specific SNVs for liver do show overlap between FF and FFPE (representing 13% and 16% of tumor-specific FF and FFPE SNVs respectively, see S3 Table). A selection of the annotated variants is shown in Table 3, and the complete list of annotated variants for colon and liver tissues is available as S4 Table. A number of variants overlap with the COSMIC database and are found in well-established tumor-related biological pathways. For instance, the KRAS, PTEN and APC genes are well-established tumor drivers in many different tumor types, including colon cancer [2]. We selected the top 5 mutated genes in liver (TERT, CTNNB1, TP53, ALB, ARID1A, [41]) and top 4 mutated genes in colon (APC, TP53, SYNE1, PIK3CA, [2]) cancer according to two recent studies and analyzed the mean coverage in FF, FFPE and for the three FFPE extraction kits for all colon and liver tumor samples (S5 Table). The average coverage values for all genes is greater than 30X for all genes in FF and FFPE samples, excepted for PIK3CA in FFPE samples. Interestingly, average coverage values are lower in FFPE compared to FF samples in many cases, and the Maxwell kit has lower average cover than the other kits, although there are exceptions to this tendency (e.g. TP53 for colon and liver samples, TERT and ARID1A for liver samples).

**Table 3:** Selection of annotated tumor-specific variants found in common between FF and FFPE colon samples. Chr: chromosome number. Position: position of the variant on the chromosome. Ns: number of samples in which the variant was found. COSMIC ID: COSMIC database identification code. Gene Symbol: HGNC gene symbol. Pathways: selection of KEGG or REACTOME pathways in which the gene is involved. FFV: mean variant allelic frequency (%) for FF samples. FFD: mean read depth for the position for FF samples (coverage). FEV: mean variant allelic frequency (%) for FFPE samples. FED: mean read depth for the position for FFPE samples (coverage).

Chr	Position	Ns	COSMIC ID	Gene Symbol	Pathways	FFV	FFD	FEV	FED
chr1	228109626	10	COSM4389123	WNT9A	WNT signaling pathway	24	208	22	428
					Basal cell carcinoma				
					Melanogenesis				
					Pathways in cancer				
					Hedgehog signaling pathway				
chr2	231867435	10	COSM5499569	SPATA3		21	116	26	145
chr2	238990381	9	COSM5471498	UBE2F-SCLY	Selenoaminoacid metabolism	23	153	22	226
			COSM5471497	SCLY					
chr3	98188932	10	COSM262658	OR5K1	Olfactory transduction	22	166	19	85
chr5	112175594	9	COSM19705	APC	WNT signaling pathway	21	142	22	73
				CTC-554D6.1	Endometrial cancer				
					Pathways in cancer				
					Colorectal cancer				
					Basal cell carcinoma				
chr5	140432413	7	COSM125200	PCDHB1	Signaling by Rho GTPases	24	79	25	54
chr6	36982783	10	COSM3076428	FGD2		22	130	18	211
chr10	50315817	10	COSM259693	VSTM4		22	180	21	286
			COSM259692						
chr10	89692910	10	COSM5032	PTEN	Pathways in cancer	31	153	31	81
					Tight junction				
					Prostate cancer				
					PI signaling system				
					Melanoma, Glioma				
					P53 signaling pathway				
chr11	64083221	3	COSM300694	TRMT112	Peroxisome	15	60	10	120
				ESRRA	Nuclear receptor transcription				
				PRDX5	Generic transcription				
chr11	76796018	3	COSM4592217	CAPN5		25	88	27	178
chr12	25398284	9	COSM1140133	KRAS	Pathways in cancer	19	88	20	61
			COSM49168		Prostate cancer				
			COSM520		Endometrial cancer				
					Acute myeloid leukemia				
					Non small cell lung cancer				
					Glioma, Thyroid cancer				
					Colorectal cancer				
					ERBB signaling pathway				
					VEGF signaling pathway				
chr13	111109670	3		COL4A2-AS2	ECM receptor interaction	48	169	48	212
				COL4A2	Pathways in cancer				
					Small cell lung cancer				
					Focal adhesion				
chr20	62076690	9	COSM2932051	KCNQ2,	Developmental biology	23	112	19	190
			COSM2932050	RP11-358D14.2	Potassium channels				
			COSM2932052	RP11-358D14.2	Neuronal system				
			COSM4100400						
chrX	37028002	10	COSM1319445	FAM47C		46	74	45	95
			COSM1319446						

## Discussion

In this study, we compared DNA samples from FF and FFPE commercial tissue samples, using whole-exome high-throughput sequencing. We systematically analyzed coverage indicators and performed variant analysis between FF and FFPE samples, and we also analyzed the influence of three different FFPE DNA extraction kits by comparing matched pairs of FF and FFPE samples.

Our results show that the quality of the DNA extracts, as measured by the DNA integrity number (DIN), is significantly lower in FFPE samples, which indicates more degraded DNA molecules. This result is expected, since it is a well-established fact that the FFPE process contributes to fragmentation, cross-linking and chemical modifications of FFPE derived nucleic acids [11, 12]. Within the FFPE samples, we observe a limited but nevertheless statistically significant difference in DIN values between the three kits, with the Maxwell kit having the lowest median DIN value. The DNA fragment length in the libraries is dramatically lower in FFPE samples compared to FF (almost 20 times smaller) and we also found a significant but much less important difference in fragment length values for the three extraction kits, with the Maxwell kit having the smallest fragment length.

The coverage analysis shows a significant difference between FF and FFPE samples for multiple indicators, with a lower percentage of positions having a coverage greater than or equal to 30X, a lower median coverage value, a higher percentage of duplicated reads and a slightly lower percentage of reads mapping outside the target regions in FFPE samples. Regarding the three FFPE extraction kits we observe moderated but significant differences for coverage indicators. The GeneRead and QIAamp are quite similar in terms of all the coverage statistics we have monitored, while the Maxwell kit has lower values for median coverage and percentage of positions having a coverage greater than or equal to 30x and a higher percentage of reads mapping outside the target regions, but on the other hand has a lower percentage of duplicated reads. Note that some metrics can be difficult to interpret together. For instance, the percentage of duplicated reads is higher for sampled treated with the Generead kit compared to Maxwell, but at the same time the coverage is higher for GeneRead samples compared to Maxwell.

As could be expected, these results indicate a lower coverage quality in FFPE samples. We find also a significant effect of the extraction kit protocols we have tried on the coverage quality metrics. However, it is worth noting that the minimum value for the percentage of positions having a coverage of 30X or more for all FFPE samples is 94%, which is well above our usual quality threshold of at least 80% applied for exome sequencing studies (see for instance [4–7]). The same applies for the percentage of duplicated reads, where the maximum values for all FFPE samples is 19% while our quality threshold is at most 25%. On the other hand, for the median coverage indicator, there are a few samples below our threshold of at least 60X, but still 86% of the samples are above this criteria. Taken together, these results show that the FFPE samples have lower coverage quality and that we can detect rather small but significant differences between the three extraction methods we have analyzed, but the resulting sequences are above usual quality standards for whole-exome sequencing.

For the variant analysis, our results detect a significant but small decrease in the number of SNVs or INDELs called in the FFPE compared to the FF sample pairs. However, the number of common variants between FF and FFPE pairs is very high in all cases (minimum 86% of the total number of SNVs), and the percentage of concordance for the common positions is also very high (99.98% mininum for SNVs). These results show that variant calling results in FFPE samples are highly similar to FF samples. Consistent with this global result, our analysis of tumor-specific variants found in both FF and FFPE samples shows that a large number of them are potentially impacting various genes and biological pathways relevant to cancer. Our results are concordant with previous studies related to whole-exome sequencing and other forms of high-throughput sequencing for FFPE samples [18–20, 22, 23]. Although we found a high percentage of concordance for variants detection between FF and FFPE samples, we detected limited but significant variations in the total number of variants difference between the three FFPE extraction kits, with a higher decrease in variants for FFPE samples extracted with the Maxwell extraction kit that can reach up to 10% of the total number of variants for SNVs.

The tumor-specific variants analysis found several candidates that overlap with knownn and well-established COSMIC variants, for genes that are found in canonical tumor-related biological pathways. For instance, genes such as KRAS, PTEN and APC are well-known tumor drivers in several types of cancer. The number of tumor-specific variants we found in FF and FFPE samples, as well as the proportion of those variants that are common between FF and FFPE are similar to values found in other exome based studies [22]. The relative low values of the common tumor-specific variants between FF and FFPE samples illustrate the feasability as well as the challenges associated with the analysis of concordant somatic mutation calls between FF and FFPE samples.

We found a significant but limited increased rate of C.G > T.A substitutions in FFPE samples (≈ 1%) compared to FF, and also a significant but very limited C.G > T.A substitution rate variations between the three FFPE extraction methods (maximum ≈ 0.01% between the GeneRead and Maxwell kits). C.G > T.A changes artifacts are caused by a chemical reaction called cytosine deamination. This change can happen spontaneously *in vivo* and is corrected by intracellular enzymes uracil DNA glycosylase (UDG) and 5-methylcytosine DNA glycosylase (with the latter repairing specifically changes occurring at CpG dinucleotides sites) [42, 43]. Formalin fixation has been reported to play an important deamination role [19, 39, 44, 45], but other factors such as UV irradiation, pH, hypoxia and heat can also have an effect [46–48]. In order to reduce deamination linked artifacts in FFPE, researchers have tried to measure the benefits of adding a pre-treatment step with UDG, which seems to be effective, with a more important effect on older specimens [39, 40, 44]. In fact, the GeneRead kit includes UDG precisely in order to repair this type of artifact. On our FFPE samples, the GeneRead kit has a significant but very limited effect on on the C.G > T.A substitution rate compared to the other kits, which might be due to the fact that the samples used in this study are only maximum two years old, whereas UDG effects has been shown to be more efficient in older specimens.

To summarize our results on the three FFPE DNA extraction kits we have analyzed in this study, we compiled all the coverage and variant calling indicators in Table 4. These indicators are of course important, but we also included indicators related to the usage of the extraction kits at the bench, more especially how easy or practical they are in the perspective of using them in projects where large numbers of samples will be processed. Although such indicators clearly do not directly affect the quality of the results, they might be important secondary indicators of high practical importance when choosing an extraction kit for a large-scale project. We also included in the table qualitative rankings (from one to three stars) indicating how the three kits performed relative to each other for all the indicators with the samples included in this study. Globally, Table 4 shows that the three kits performed very well regarding the concordance of variant calling between FF and FFPE sample pairs. However, when looking at the various indicators of Table 4, we can see that the Qiagen kits (GeneRead, QIAamp) perform better on several indicators compared to the Maxwell^™^ kit on the samples used in this study, but it is also worth noticing that this kit has several technical and practical advantages, such as a cassette system that is easy to use and a large number of samples that can be processed per run. Few studies have analyzed the effect of DNA extraction systems and kits. Heydt et al. [28] included a Maxwell 16 DNA extraction system (an older version of the machine we used in this study) in their analysis of five DNA extraction systems on FFPE samples, and concluded that this system and associated extraction kit was giving better results in terms of DNA concentration. However, a previous study focusing on the same Maxwell 16 system found a higher DNA concentration using another extraction kit [49]. Those two studies used a different Maxwell automated extraction system and associated FFPE extraction kit, which may explain the different results.

**Table 4:** Coverage, variant calling and technical quality indicators for the three FFPE DNA extraction kits. Values are given for quantitative indicators. The number of stars between brackets (one, two or three) indicate the relative ranking of a given kit for the indicator and for the samples we have analyzed in this study. Technical indicators (qualitative and quantitative) describe how easy or practical it is to use the kits, especially with the aim of analyzing large number of samples, based on our experience in this study.

	GeneRead	Maxwell	QIAamp
**Coverage and variants indicators**			
DNA Integrity Number median value	2.35 (**)	1.75 (*)	3.3 (***)
DNA fragment length in bp	1424 (**)	988 (*)	1622 (***)
Median percentage of positions with coverage ≥ 30X ^1^	97.6 (***)	95.5 (*)	97.7 (***)
Median coverage values ^1^	76X (**)	60X (*)	87.5X (***)
Percentage of duplicated reads ^1^	15.5 (*)	7.4 (***)	12.2 (*)
Percentage of reads mapping outside target regions^1^	19.6 (**)	21.4 (*)	19.1 (**)
C.T > G.A conversion rate in SNV calls	0.372	0.374	0.372
Median value for the variant difference FF/FFPE ^2^	214 (***)	3367 (*)	667 (**)
Median value for the percentage of concordance FF/FFPE ^3^	99.9 (***)	99.9 (***)	99.9 (***)
**Technical indicators for the extraction process**			
Purification technique easiness ^4^	(*)	(***)	(*)
Possibility of elution step optimization ^5^	(**)		(**)
Output tube format and transfer ^6^	(***)	(*)	(***)
Max number of samples per run	12 (**)	16 (***)	12 (**)
Input material quantity (nb of 10 *μ*m tissue slices / extraction) ^7^	1 (*)	1 to 16 (***)	1 to 8 (**)

^1^ The values are calculated on bam files normalized to 80M reads.

^2^ The value corresponds to the absolute value of the difference between the number of variants in FF versus FFPE sample pairs.

^3^ The percentage of concordance is determined on the common variants positions between FF and FFPE sample pairs.

^4^ Purification for the Maxwell kit is based on magnetic beads and cassettes, GeneRead and QIAamp kits use columns which are more time consuming to manipulate.

^5^ The elution volume is fixed for the Maxwell kit, leaving no room for optimization, while with the QIAGEN kits, it is possible to optimize the final concentration by playing with the elution volume and/or to warm up the elution buffer to increase efficiency.

^6^ For the Maxwell kit, the format of the output tube (0.5 ml) is not practical, and there are magnetic beads residues in the tubes, necessitating a transfer in a more adapted tube for further processing. These problems are not present for the QIAGEN kits.

^7^ The higher the number of tissue slices per extraction, the more flexible it is to obtain enough material for sequencing.

In summary, our results demonstrate that high-throughput whole-exome analysis of variants on FFPE samples have high quality of coverage and a very high percentage of variants concordance when compared to paired FF samples. Limited but significant variations in coverage and variant calling indicators can be detected for the three different FFPE DNA extraction kits on the samples included in this study. The values of all the indicators are above usually accepted thresholds, but the differences suggest that the selection of a kit for a large scale project (e.g. precision medicine) should be done with care and thorough testing beforehand.

## Supporting information

S1 Table Samples description. (PDF)

S2 Table FF / FFPE sample pairs description. (PDF)

S3 Table Tumor specific SNV counts and percentages. (PDF)

S4 Table Excel file listing all the annotated tumor-specific SNVs for colon and liver tissue samples. (XLSX)

S5 Table Average coverage values for the top mutated genes in colon and liver cancer. (PDF)
