# Supplementary material for: Performance comparison of three DNA extraction kits on human whole-exome data from formalin-fixed paraffin-embedded normal and tumor samples
Source: PLoS One. 2018 Apr 5;13(4):e0195471. doi: 10.1371/journal.pone.0195471 (PMC5886566; doi:10.1371/journal.pone.0195471)
Supplement: S1 Table — (PDF) [file pone.0195471.s001.pdf]

Performance comparison of three dna extraction kits on human whole-exome formalin-fixed paraffin-embedded samples
S1 supplementary table

2018

S1 Table. Samples description. FFPE and FF samples were ordered from company AMS BIOTECHNOLOGY EUROPE Ltd. We included tissues from two different organs (liver and colon). For each tissue, all samples come from the same individual and include both normal and tumor samples from fresh-frozen and formalin-fixed, paraffin-embedded tissues.

**Sample**	**Tissue source**	**Extraction method**	**FF/FFPE**
B00GXDN	liver tumoral	GeneRead	FFPE
B00GXDO	liver tumoral	GeneRead	FFPE
B00GXDP	liver tumoral	GeneRead	FFPE
B00GXDQ	liver normal	GeneRead	FFPE
B00GXDR	liver normal	GeneRead	FFPE
B00GXDS	liver normal	GeneRead	FFPE
B00GXHK	colon tumoral	GeneRead	FFPE
B00GXHL	colon tumoral	GeneRead	FFPE
B00GXHH	colon normal	GeneRead	FFPE
B00GXHI	colon normal	GeneRead	FFPE
B00GXCQ	liver tumoral	QIAamp DNA FFPE	FFPE
B00GXCR	liver tumoral	QIAamp DNA FFPE	FFPE
B00GXCS	liver normal	QIAamp DNA FFPE	FFPE
B00GXCT	liver normal	QIAamp DNA FFPE	FFPE
B00GXKT	colon tumoral	QIAamp DNA FFPE	FFPE
B00GXKU	colon tumoral	QIAamp DNA FFPE	FFPE
B00GXKW	colon normal	QIAamp DNA FFPE	FFPE
B00GXKX	colon normal	QIAamp DNA FFPE	FFPE
B00GXDH	liver tumoral	QIAamp DNAMicro	FF
B00GXDI	liver tumoral	QIAamp DNAMicro	FF
B00GXDL	liver normal	QIAamp DNAMicro	FF
B00GXDM	liver normal	QIAamp DNAMicro	FF
B00GXJ0	colon tumoral	QIAamp DNAMicro	FF
B00GXJ1	colon tumoral	QIAamp DNAMicro	FF
B00GXJ3	colon normal	QIAamp DNAMicro	FF
B00GXJ4	colon normal	QIAamp DNAMicro	FF
B00GXL9	colon tumoral	Maxwell frozen tissue	FF
B00GXLB	colon tumoral	Maxwell frozen tissue	FF
B00GXLE	colon normal	Maxwell frozen tissue	FF
B00GXLF	colon normal	Maxwell frozen tissue	FF
B00GXLH	liver tumoral	Maxwell frozen tissue	FF
B00GXLJ	liver tumoral	Maxwell frozen tissue	FF
B00GXLN	liver normal	Maxwell frozen tissue	FF
B00GXLO	liver normal	Maxwell frozen tissue	FF
B00GXLQ	colon tumoral	Maxwell FFPE	FFPE
B00GXLR	colon tumoral	Maxwell FFPE	FFPE
B00GXLU	colon normal	Maxwell FFPE	FFPE
B00GXLV	colon normal	Maxwell FFPE	FFPE
B00GXLY	liver tumoral	Maxwell FFPE	FFPE
B00GXLZ	liver tumoral	Maxwell FFPE	FFPE
B00GXM2	liver normal	Maxwell FFPE	FFPE
B00GXM5	liver normal	Maxwell FFPE	FFPE
