# Supplementary material for: Performance comparison of three DNA extraction kits on human whole-exome data from formalin-fixed paraffin-embedded normal and tumor samples
Source: PLoS One. 2018 Apr 5;13(4):e0195471. doi: 10.1371/journal.pone.0195471 (PMC5886566; doi:10.1371/journal.pone.0195471)
Supplement: S2 Table — (PDF) [file pone.0195471.s002.pdf]

Performance comparison of three dna extraction kits on human whole-exome formalin-fixed paraffin-embedded samples Supplementary table S2

2018

S2 Table. FF and FFPE sample pairs used for the analysis. The pair 26 was not used in the analysis due to a low number of reads after mapping to the human genome in one of the samples (B00GXLO, nb reads $< 80M$).

	**FF**		**FFPE**	
**pair ID**	**barcode**	**Sample type**	**barcode**	**Sample type**
1	B00GXDH	FF liver tumoral	B00GXCQ	FFPE liver tumoral
2	B00GXDI	FF liver tumoral	B00GXCR	FFPE liver tumoral
3	B00GXDL	FF liver normal	B00GXCS	FFPE liver normal
4	B00GXDM	FF liver normal	B00GXCT	FFPE liver normal
5	B00GXJ0	FF colon tumoral	B00GXKT	FFPE colon tumoral
6	B00GXJ1	FF colon tumoral	B00GXKU	FFPE colon tumoral
7	B00GXJ3	FF colon normal	B00GXKW	FFPE colon normal
8	B00GXJ4	FF colon normal	B00GXKX	FFPE colon normal
9	B00GXDH	FF liver tumoral	B00GXDN	FFPE liver tumoral
10	B00GXDI	FF liver tumoral	B00GXDO	FFPE liver tumoral
11	B00GXDI	FF liver tumoral	B00GXDP	FFPE liver tumoral
12	B00GXDL	FF liver normal	B00GXDQ	FFPE liver normal
13	B00GXDM	FF liver normal	B00GXDR	FFPE liver normal
14	B00GXDM	FF liver normal	B00GXDS	FFPE liver normal
15	B00GXJ0	FF colon tumoral	B00GXHK	FFPE colon tumoral
16	B00GXJ1	FF colon tumoral	B00GXHL	FFPE colon tumoral
17	B00GXJ3	FF colon normal	B00GXHH	FFPE colon normal
18	B00GXJ4	FF colon normal	B00GXHI	FFPE colon normal
19	B00GXL9	FF colon tumoral	B00GXLQ	FFPE colon tumoral
20	B00GXLB	FF colon tumoral	B00GXLR	FFPE colon tumoral
21	B00GXLE	FF colon normal	B00GXLU	FFPE colon normal
22	B00GXLF	FF colon normal	B00GXLV	FFPE colon normal
23	B00GXLH	FF liver tumoral	B00GXLY	FFPE liver tumoral
24	B00GXLJ	FF liver tumoral	B00GXLZ	FFPE liver tumoral
25	B00GXLN	FF liver normal	B00GXM2	FFPE liver normal
26	B00GXLO	FF liver normal	B00GXM5	FFPE liver normal
