# Supplementary material for: Performance comparison of three DNA extraction kits on human whole-exome data from formalin-fixed paraffin-embedded normal and tumor samples
Source: PLoS One. 2018 Apr 5;13(4):e0195471. doi: 10.1371/journal.pone.0195471 (PMC5886566; doi:10.1371/journal.pone.0195471)
Supplement: S3 Table — (PDF) [file pone.0195471.s003.pdf]

Performance comparison of three dna extraction kits on human whole-exome formalin-fixed paraffin-embedded samples
Supplementary table S3

2018

S3 Table. Tumor specific SNVs counts and percentages. Tissue: tissue source type; NbV FF N: Nb of unique variants in all FF normal samples; NbV FF TS: Nb of unique variants in all FF samples that are tumor specific; NbV FFPE N: Nb of unique variants in all FFPE normal samples; NbV FFPE TS: Nb of unique variants in all FFPE samples that are tumor specific; Nb CV: Nb of variants that are common between tumor specific variants for FF and FFPE samples; P FF: percentage of FF and FFPE common variants relative to the number of FF tumor specific variants (Nb CV / NbV FF TS); P FFPE: percentage of FF and FFPE common variants relative to the number of FFPE tumor specific variants (Nb CV / NbV FFPE TS).

Tissue	NbV FF N	NbV FF TS	NbV FFPE N	NbV FFPE TS	Nb CV	P FF	P FFPE
Liver	41903	394	42204	333	53	13%	16%
Colon	41979	436	42183	458	165	38%	36%
