# Supplementary material for: Performance comparison of three DNA extraction kits on human whole-exome data from formalin-fixed paraffin-embedded normal and tumor samples
Source: PLoS One. 2018 Apr 5;13(4):e0195471. doi: 10.1371/journal.pone.0195471 (PMC5886566; doi:10.1371/journal.pone.0195471)
Supplement: S5 Table — (PDF) [file pone.0195471.s005.pdf]

Performance comparison of three dna extraction kits on human whole-exome formalin-fixed paraffin-embedded samples
Supplementary table S5

2018

S5 table. Average coverage values for the top mutated genes in colon and liver cancer. The columns indicate average coverage values per gene for FF and FFPE samples, and also for each extraction kit of the FFPE samples (GeneRead, Maxwell and QIAamp).

**Gene Symbol**	**FF**	**FFPE**	**GeneRead**	**Maxwell**	**QIAamp**
Colon tumor samples					
APC	95.33	53.77	65.87	27.09	68.33
TP53	35.44	44.72	34.69	58.28	41.20
SYNE1	68.79	50.87	48.57	41.58	62.47
PIK3CA	40.69	22	31.09	11.31	23.59
Liver tumor samples					
TERT	75.22	125.93	136.31	158.08	78.22
CTNNB1	44.93	31.80	31.17	20.32	44.23
TP53	36.45	40.46	30.60	56.21	39.48
ALB	90.11	63.85	72.41	27.48	87.37
ARID1A	85.16	94.04	73.46	125.33	93.61
